# Characterization and Oral Delivery of Proinsulin-Transferrin Fusion Protein Expressed Using ExpressTec

**DOI:** 10.3390/ijms19020378

**Published:** 2018-01-26

**Authors:** Yu-Sheng Chen, Jennica L. Zaro, Deshui Zhang, Ning Huang, Andrew Simon, Wei-Chiang Shen

**Affiliations:** 1 Department of Pharmacology and Pharmaceutical Sciences, University of Southern California School of Pharmacy, Los Angeles, CA 90089, USA; yushengc@usc.edu (Y.-S.C.); jZaro@westcoastuniversity.edu (J.L.Z.); 2 Department of Pharmaceutical Sciences, West Coast University School of Pharmacy, Los Angeles, CA 90004, USA; 3 Ventria Bioscience Inc., 2718 Industrial Drive, Junction City, KS 66441, USA; deshuizhang18@gmail.com (D.Z.); nhuang2010@gmail.com (N.H.); asimon@ventria.com (A.S.)

**Keywords:** transgenic rice, proinsulin, transferrin, fusion protein, diabetes, oral delivery

## Abstract

Proinsulin-transferrin fusion protein (ProINS-Tf) has been designed and successfully expressed from the mammalian HEK293 cells (HEK-ProINS-Tf). It was found that HEK-ProINS-Tf could be converted into an activated form in the liver. Furthermore, HEK-ProINS-Tf was demonstrated as an extra-long acting insulin analogue with liver-specific insulin action in streptozotocin (STZ)-induced type 1 diabetic mice. However, due to the low production yield from transfected HEK293 cells, there are other interesting features, including the oral bioavailability, which have not been fully explored and characterized. To improve the protein production yield, an alternative protein expression system, ExpressTec using transgenic rice (*Oryza sativa* L.), was used. The intact and active rice-derived ProINS-Tf (ExpressTec-ProINS-Tf) was successfully expressed from the transgenic rice expression system. Our results suggested that, although the insulin-like bioactivity of ExpressTec-ProINS-Tf was slightly lower in vitro, its potency of in vivo blood glucose control was considerably stronger than that of HEK-ProINS-Tf. The oral delivery studies in type 1 diabetic mice demonstrated a prolonged control of blood glucose to near-normal levels after oral administration of ExpressTec-ProINS-Tf. Results in this report suggest that ExpressTec-ProINS-Tf is a promising insulin analog with advantages including low cost, prolonged and liver targeting effects, and most importantly, oral bioactivity.

## 1. Introduction

Proinsulin-transferrin fusion protein (ProINS-Tf), which has been successfully expressed from HEK293 mammalian cells (HEK-ProINS-Tf), is a novel long-acting insulin analogue with liver-preferential bioactivity [1]. It has been demonstrated that the proinsulin domain of HEK-ProINS-Tf can be intracellularly converted into an active form via Tf receptor (TfR)-mediated endocytosis and recycling pathways in H4IIE hepatoma cells [2] and in mouse liver tissue slices [3]. The activated ProINS-Tf exhibits a prolonged effect on the phosphorylation of Akt protein in H4IIE cells [2] and on the lowering of blood glucose levels in a type 1 diabetic mouse model under fasting conditions, where the liver plays a predominant role in controling blood glucose levels [1,2,3]. The liver has long been considered as the major organ in glucose homeostasis [4] and is the target organ of endogenous insulin action under physiological conditions [5,6]. Therefore, ProINS-Tf is considered an ideal insulin analog for the treatment of insulin-deficient diabetes due to its liver-targeted effects [5,6]. To further investigate the potential applications of ProINS-Tf in diabetes treatment, alternative expression systems were explored in this study aimed at achieving a high production yield for this recombinant fusion protein.

Transgenic plant expression systems are attractive options for manufacturing recombinant proteins due to the cost effectiveness and high scale-up capacity [7,8]. It was recently demonstrated that a Tf-based fusion protein, exendin-4-Tf, could be produced in transgenic tobacco plants with promising potential for oral delivery [9]. In this report, the ExpressTec (Ventria Bioscience Inc., Junction City, KS, USA) was chosen as an alternative expression system for ProINS-Tf because it has successfully produced human transferrin (hTf) with a high production yield and low production cost [10]. According to previous reports, rice-derived hTf shares structural and functional similarities with native hTf [11]. Therefore, one of the aims of this study was to determine if ProINS-Tf could be successfully expressed in rice, with a similar biological characterization and activity as the HEK293 cell-produced counterpart. In addition, Tf has been recombinantly fused with several protein therapeutics, such as growth hormone and granulocyte-colony stimulating factor, to increase their oral bioactivities [12,13]. With the advantage of high production yield, the oral bioactivity of the ExpressTec-ProINS-Tf was also investigated in streptozotocin (STZ)-induced type 1 diabetic mice.

## 2. Results

### 2.1. Expression, Extraction and Purification of ExpressTec-Proinsulin-Transferrin Fusion Protein (ExpressTec-ProINS-Tf)

**Figure 1:**
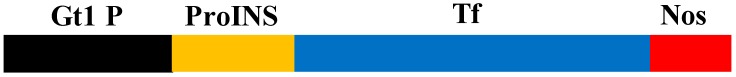
Diagram of the plasmid construct for the expression of proinsulin-transferrin fusion protein (ProINS-Tf). Gt1 P, glutelin promoter; ProINS, the codon-optimized proinsulin gene; Tf, the codon-optimized human transferrin gene; Nos, nopaline synthase terminator.

To obtain high level expression of the target protein in rice, the genetic codons of the fusion gene encoding ProINS-Tf fusion protein were optimized using a preferred algorithm However, the amino acid sequences of both ProINS and Tf remained unchanged because of the codon degeneracy. Then, the two synthetic and codon-optimized nucleotide sequences encoding ProINS protein and Tf protein were fused in frame and placed under the control of the upstream rice glutelin gene promoter (Gt1) resulting in the construction of the plasmid shown in Figure 1. The plasmid construct was verified via DNA sequencing with full coverage on both strands.

Rice genetic transformation was performed as previously described [10]. Briefly, the linear expression cassette DNA fragments, comprising the region from promoter to terminator (without the superfluous backbone plasmid sequence) of both the gene of interest and a selectable marker gene, were used for microprojectile bombardment-mediated transformation of embryonic calli induced from the mature seeds of rice (*O. sativa)*. Regenerated transgenic seedlings were confirmed through PCR analysis with primers specific to the ProINS-Tf gene.

Protein expression screening analysis was performed on the first generation of transgenic rice seeds (R1) by immune assays of seed protein extracts with both anti-human proinsulin and anti-human transferrin antibodies as probes. Positive transgenic events were selected for high level expression yields of ExpressTec-ProINS-Tf. The sodium dodecyl sulfate polyacrylamide gel electrophoresis (SDS–PAGE) analysis of transgenic events with high level expression of ExpressTec-ProINS-Tf revealed a predominant protein band corresponding to the calculated molecular weight of ProINS-Tf (79 kDa) in positive transgenic seeds but not in the wild-type rice seeds (data not shown).

### 2.2. Molecular Characterization of ExpressTec-ProINS-Tf

**Figure 2:**
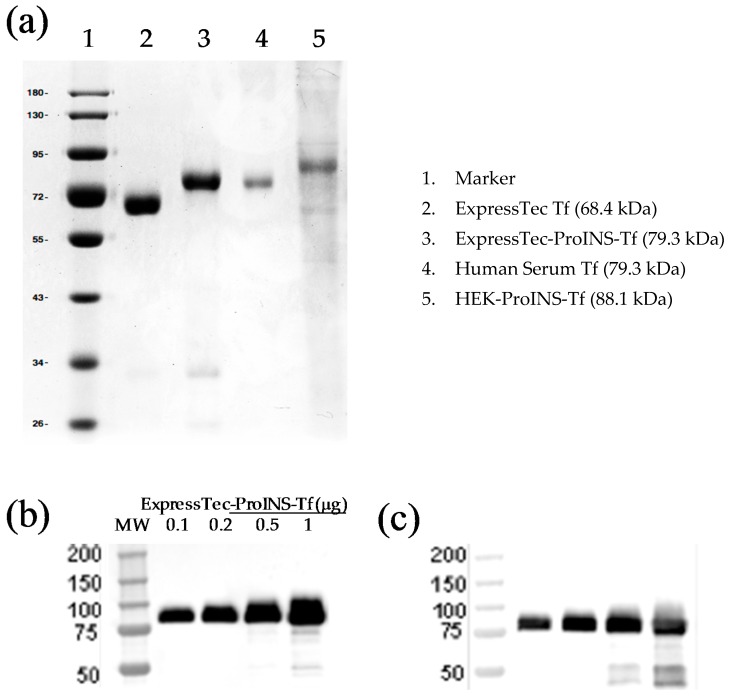
Molecular characterization of ExpressTec ProINS-Tf. (**a**) 10% SDS-PAGE followed by Coomassie blue staining. The indicated molecular weights were estimated by Image Lab^TM^ Software (**b**) Western blot probed with anti-ProINS antibody. 0.1, 0.2, 0.5 and 1 μg of ExpressTec-ProINS-Tf were loaded, respectively. (**c**) Western blot probed with anti-Tf antibody. 0.1, 0.2, 0.5 and 1 μg of ExpressTec-ProINS-Tf were loaded, respectively.

SDS-PAGE followed by Coomassie blue staining and Western blotting was applied to determine the authenticity of the purified ExpressTec-ProINS-Tf from rice seeds. The result of Coomassie blue staining (Figure 2a) showed that the estimated molecular weight of ProINS-Tf fusion protein (79.3 kDa) was close to the expected value. The minor protein band (34 kDa) in purified ExpressTec-ProINS-Tf (Figure 2a) corresponded to an impurity protein from the host cells, which should not affect the structure and function of ProINS-Tf fusion protein. The estimated molecular weight of ExpressTec-ProINS-Tf (79.3 kDa) was higher than the one of rice-derived Tf (68.4 kDa) but lower than HEK-ProINS-Tf’s molecular weight (88.1 kDa). The lower molecular weight suggested the absence of glycosylation of ExpressTec-ProINS-Tf, a finding which is consistent with that in rice-derived hTf [10]. The results of Western blotting (Figure 2b,c) showed that the ExpressTec-ProINS-Tf molecule could be recognized by both anti-Tf and anti-ProINS antibodies. Both results confirmed the structural features of ExpressTec-ProINS-Tf and demonstrated that ProINS-Tf fusion protein was successfully expressed from the rice (*O. sativa* L.) expression system.

### 2.3. In Vitro Comparisons between ExpressTec-ProINS-Tf and HEK293-ProINS-Tf

**Figure 3:**
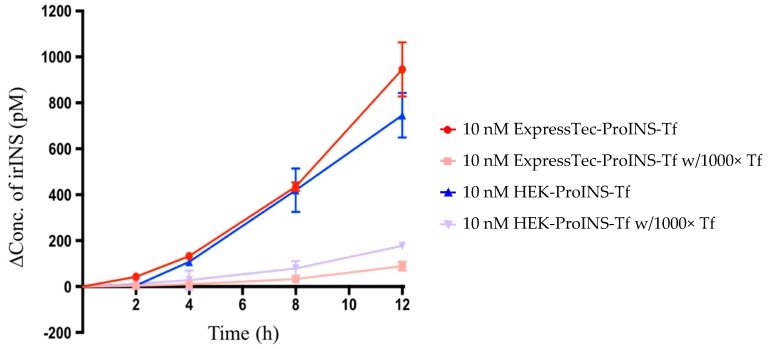
TfR-mediated conversion of ExpressTec-ProINS-Tf in H4IIE cells. H4IIE cells were treated with 10 nM of ExpressTec-ProINS-Tf or HEK-ProINS-Tf in the presence or absence of 1000-fold excess apo-Tf at 37 °C. At the specific time-points, media were collected and insulin concentrations (mean ± SD, *n* = 3) were measured using INS-specific RIA. The concentration of irINS-Tf was calculated using human insulin as a standard curve from 12 to 1200 pM according to the manufacturer’s instructions.

In order to test the conversion of ExpressTec-ProINS-Tf to the activated insulin form, the conditioned medium from H4IIE cells treated with the fusion protein was analyzed by INS-specific radioimmunoassay (RIA), which recognizes immuno-reactive insulin, but not proinsulin. In the conversion study shown in Figure 3, the increased concentration of RIA-detected insulin concentration demonstrated that ExpressTec-ProINS-Tf exhibited conversion characteristics similar to HEK-ProINS-Tf. Also, the 12 h conversion of ExpressTec-ProINS-Tf (9.46 ± 1.17%) was slightly higher than that of HEK-ProINS-Tf (7.46 ± 0.97%) in H4IIE cell culture. In the presence of excess apo-Tf, the conversion of both ExpressTec-ProINS-Tf and HEK-ProINS-Tf were significantly blocked. This result suggested that the conversion of ExpressTec-ProINS-Tf was controlled by Tf receptor-mediated endocytosis and recycling pathways, as previously established for HEK-ProINS-Tf [1].

**Figure 4:**
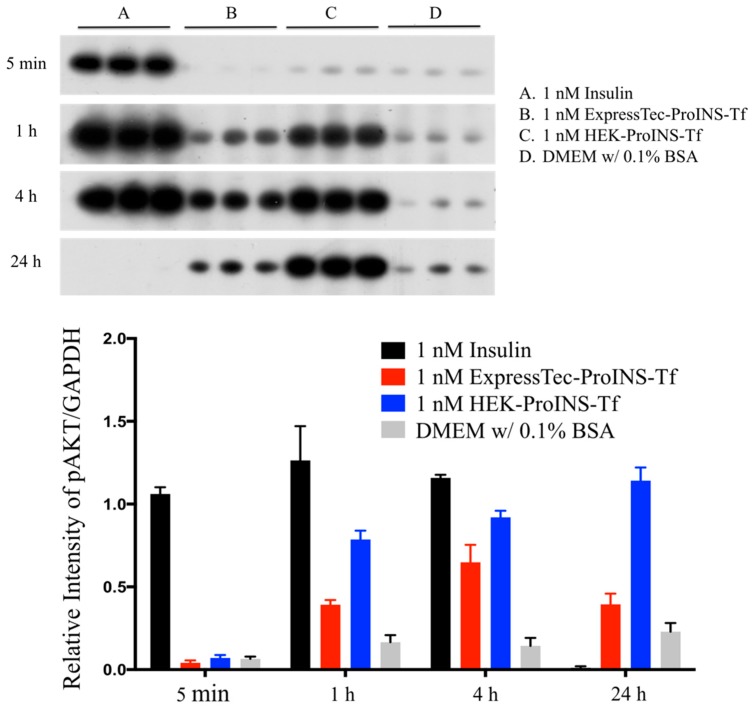
Stimulation of Akt phosphorylation in H4IIE cells. Serum-deprived H4IIE cells were treated with 1 nM of INS, ExpressTec-ProINS-Tf, HEK-ProINS-Tf or DMEM *w*/0.1% BSA at 37 °C for 5 min, 1 h, 4 h, and 24 h, respectively. After lysed with protein extraction buffer in the presence of protease and phosphatase inhibitors, 10 μg of total protein were loaded to 10% SDS-PAGE and probed with anti-phospho-Akt antibody. The data were expressed as the intensity (mean ± SD) determined using Quantity One 1-D Analysis software (Bio-Rad, Hercules, CA, USA) with immunoblot using anti-GAPDH antibody as a loading control.

To characterize the in vitro insulin-like bioactivity of different proteins, phosphorylation of Akt (pAkt) stimulated by insulin, ExpressTec-ProINS-Tf or HEK-ProINS-Tf was investigated and compared. The results from the Akt phosphorylation study (Figure 4) showed that H4IIE cells treated with 1 nM insulin exhibited a rapid onset and strong phospho-Akt signal at 5-min, 1-h and 4-h time points. The pAkt signal of the insulin treatment group disappeared at the 24-h time point. However, for both ExpressTec-ProINS-Tf and HEK-ProINS-Tf groups, the pAkt signal was initially observed at the 1-h time point and was sustained until 24 h of incubation. This lag time of Akt phosphorylation suggested ExpressTec-ProINS-Tf, just like HEK-ProINS-Tf [1], had low intrinsic activity initially, and required conversion to an active form to display its effects.

**Figure 5:**
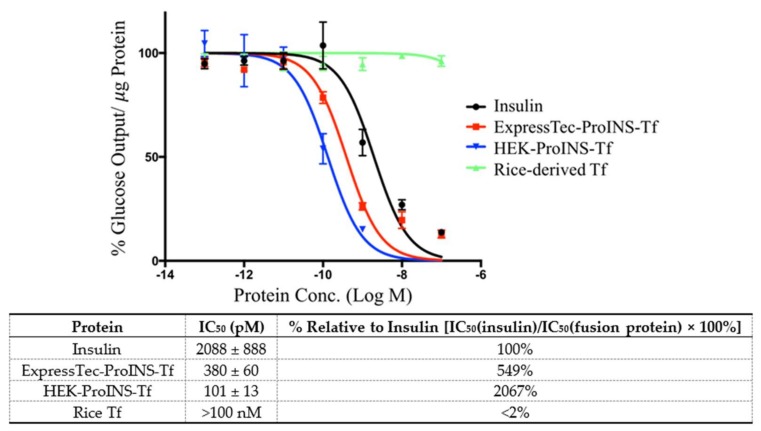
Glucose production inhibition in H4IIE cells and calculated IC_50_. H4IIE cells were treated with insulin, ExpressTec-ProINS-Tf, HEK-ProINS-Tf or rice-derived Tf with various concentrations from 0.1 to 100 nM at 37 °C for 24 h, and then incubated with glucose production medium for an additional 3 h. Glucose concentration of each sample was measured using the Amplex Red Glucose/Glucose Oxidase Kit. The result was expressed as percentage of total glucose output in the absence of any protein (*n* = 3). All data were normalized with total cell protein amounts. The indicated IC_50_ values (mean ± SD) were calculated using GraphPad Prism (GraphPad Software Inc., San Diego, CA, USA).

The inhibition effect of glucose production by a 24-h incubation of ExpressTec-ProINS-Tf was investigated and compared with that of insulin, HEK-ProINS-Tf and rice-derived Tf (Figure 5). Following a 24-h incubation, rice-derived Tf did not exert any inhibitory effect on glucose production (IC_50_ >100 nM). The inhibitory effect of ExpressTec-ProINS-Tf (IC_50_ = 380 ± 60 pM) was 5.5-fold stronger compared to that of insulin (IC_50_ = 2088 ± 888 pM) but was less than that of HEK-ProINS-Tf (IC_50_ = 101.2 ± 13.3 pM) which is about 20-fold stronger than that of insulin (Figure 5). This result, consistent with the result from the Akt phosphorylation study, suggested that ExpressTec-ProINS-Tf showed a lower in vitro insulin-like bioactivity than HEK-ProINS-Tf in H4IIE cells.

### 2.4. In Vivo Glucose-Lowering Effects following Subcutaneous Injection in Type 1 Diabetic Mice

**Figure 6:**
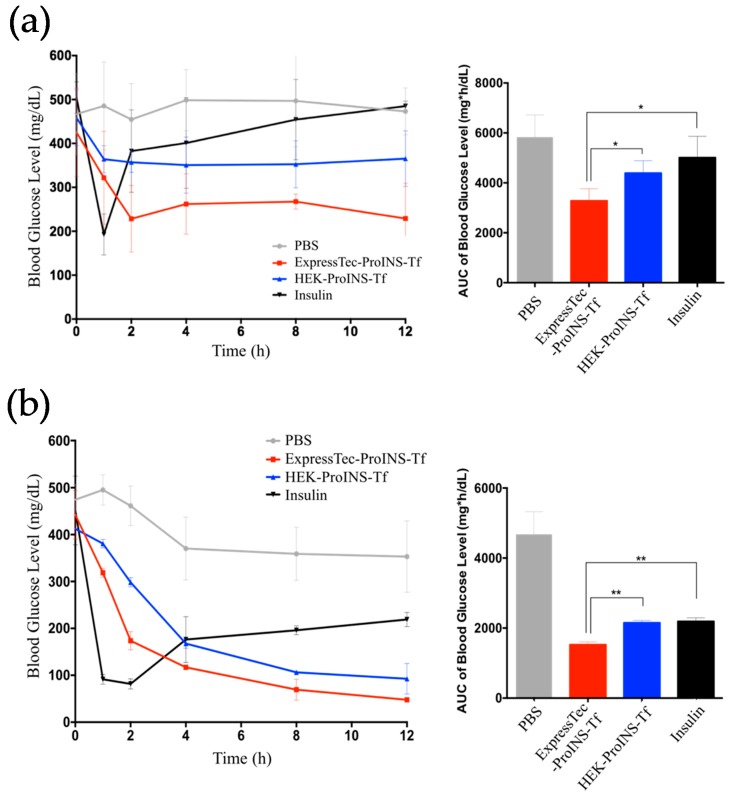
Glucose-lowering efficacy of ExpressTec-ProINS-Tf via subcutaneous injection. (**a**) STZ-induced type 1 diabetes mice under free-fed condition were subcutaneously injected with protein (22.5 nmol/kg) or PBS, and had free access to food and water during the experiment (*n* ≥ 3 per group). Blood glucose levels were monitored at indicated time points. The AUC of blood glucose level (mean ± SD) from 0-h to 12-h time points in the right panel were calculated using GraphPad Prism (GraphPad Software Inc., San Diego, CA, USA). (**b**) STZ-induced type 1 diabetic mice were fasted for 2 h before the subcutaneous injection of protein or PBS (*n* ≥ 3). Mice were kept fasted with free access to water until 12 h post injection. Blood glucose levels were monitored at the indicated time points. The AUC of blood glucose level (mean ± SD) from 0-h to 12-h time points in the right panel were calculated using GraphPad Prism (GraphPad Software Inc., San Diego, CA, USA). * *p* < 0.05; ** *p* < 0.01.

The glucose-lowering effect of subcutaneously administered ExpressTec-ProINS-Tf was tested in STZ-induced type-1 diabetic mice. For the study on free-fed mice (Figure 6a), insulin exhibited a quick onset but short duration of glucose-lowering effect. As expected, the blood glucose levels rapidly decreased within the first hour and then bounced back to the initial blood glucose level after the 2-h time point. For ExpressTec-ProINS-Tf and HEK-ProINS-Tf groups, a slower onset but sustained effect to at least 12 h post-injection was observed. The glucose levels from 4 to 12 h of ExpressTec-ProINS-Tf and HEK-ProINS-Tf were maintained at around 250 mg/dL (56% of initial level) and 350 mg/dL (78% of initial level), respectively. For the study under fasting condition, the insulin groups (Figure 6b) also showed a quick onset of blood glucose reduction, and the blood glucose levels were elevated to around 200 mg/dL after 2 h post-injection. Both ExpressTec- and HEK-ProINS-Tf-treated groups exhibited slow but long-term control of blood glucose levels, which were maintained near normal glucose levels at around 100 mg/dL. Similar to the free-fed experiment, the glucose-lowering effect of ExpressTec-ProINS-Tf was stronger than that of HEK-ProINS-Tf. Based on the calculated area under the curve (AUC) of blood glucose levels at 22.5 nmol/kg dose, ExpressTec-ProINS-Tf showed a significantly stronger effect on overall blood glucose control than insulin and HEK-ProINS-Tf.

### 2.5. In Vivo Glucose-Lowering Effects Following Oral Administration in Type 1 Diabetic Mice

**Figure 7:**
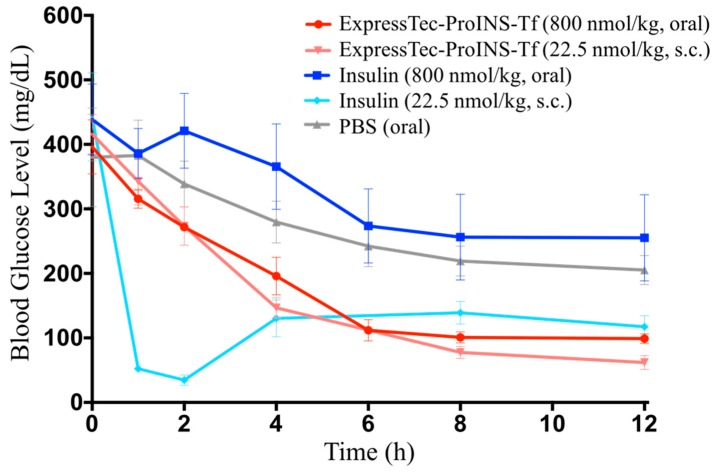
Glucose-lowering effect of ExpressTec-ProINS-Tf via oral administration. STZ-induced type 1 diabetic mice were fasted for 3 h before the oral or subcutaneous (s.c.) administration of protein at the indicated doses or PBS (*n* ≥ 3 per group). Mice were kept fasted with free access to water until 12 h post injection. Blood glucose levels were monitored at the indicated time points.

Oral glucose-lowering efficacy of ExpressTec-ProINS-Tf and insulin was also investigated in the STZ-induced type 1 diabetic mouse model in comparison to the subcutaneously injected proteins. As shown in Figure 7, the effect of subcutaneous injection of insulin or ExpressTec-ProINS-Tf (22.5 nmol/kg) on blood glucose levels was similar to the results obtained in Figure 6b. Compared to the control group (oral PBS), there is no significant decrease in blood glucose levels in STZ-induced diabetic mice after oral administration of insulin at 800 nmol/kg. However, the oral administration of ExpressTec-ProINS-Tf at 800 nmol/kg exhibited a slow but significant decrease of blood glucose levels from 4 h to at the latest time point tested (12 h). The level of blood glucose in the oral ExpressTec-ProINS-Tf group was maintained at normal blood glucose levels (~100 mg/dL) from 6 h to at least 12 h post-injection.

## 3. Discussion

The results presented in this manuscript demonstrate that the ProINS-Tf fusion protein can be successfully expressed from rice. ExpressTec-ProINS-Tf exhibited similar molecular characteristics and bioactivity as HEK-ProINS-Tf with some minor differences, possibly due to the lack of glycosylation of ExpressTec-ProINS-Tf. Furthermore, Akt phosphorylation and glucose production inhibition assays indicated that the in vitro activity of ExpressTec-ProINS-Tf was slightly lower than that of HEK-ProINS-Tf. However, its potency in in vivo blood glucose control was considerably higher than that of HEK-ProINS-Tf. This unexpected discrepancy between in vitro and in vivo effects could be explained by the potential dual receptor targeting of activated ProINS-Tf. As a bifunctional fusion protein that binds both TfR and insulin receptor (IR), the affinity toward each respective receptor of the two domains would be evaluated to determine the pharmacokinetic profile of ProINS-Tf [14]. Binding to TfR would most likely increase the plasma half-life of ProINS-Tf due to its recycling nature [15], while binding to IR would lead to the degradation of the fusion protein via the IR-mediated degradation pathway [16]. Since the binding affinity of ExpressTec-ProINS-Tf to IR is lower than that of HEK-ProINS-Tf (Figure 4 and Figure 5), it can be anticipated that ExpressTec-ProINS-Tf will exhibit a longer plasma and liver half-life and, thus, longer in vivo biological activity, compared to HEK-ProINS-Tf.

It has been shown that ExpressTec-Tf exhibits a similar TfR binding affinity and intracellular trafficking as native hTf [11]. In our studies, we also observed a similar result in the competitive TfR binding assay, indicating no significant difference in TfR affinity between HEK- and ExpressTec-ProINS-Tf. However, the lower effect of ExpressTec-ProINS-Tf on both the Akt phosphorylation (Figure 4) and the inhibition of glucose production (Figure 5) compared to HEK-ProINS-Tf in H4IIE cells suggested that the activated ExpressTec-ProINS-Tf possesses a lower binding affinity to IR than HEK-ProINS-Tf. Therefore, once converted in vivo, the activated ExpressTec-ProINS-Tf with a weaker binding affinity to IR may lead to a lower IR-mediated intracellular degradation and a higher TfR-mediated recycling in comparison to HEK-ProINS-Tf. As a result, more intracellularly activated ExpressTec-ProINS-Tf could be recycled by TfR and released to the blood circulation, to display a stronger and longer glucose-lowering effect in peripheral tissues. Also, due to a lower IR binding as evidenced by the weaker Akt phosphorylation and glucose production activity (Figure 4 and Figure 5), relatively more activated ExpressTec-ProINS-Tf could be released from liver to peripheral tissues compared to HEK-ProINS-Tf. Consequently, ExpressTec-ProINS-Tf would have a shorter onset and stronger glucose-lowering efficacy in vivo compared to HEK-ProINS-Tf, especially under the free-feeding condition, i.e., 50% versus 25% decrease of blood glucose level at 12 h (Figure 6a). Under feeding conditions, it has been shown that less than 34% of oral glucose load is stored in the liver through glycogen synthesis [17]. Therefore, the stronger glucose-lowering activity under feeding conditions supports our hypothesis that ExpressTec-ProINS-Tf exerts its effect on both the liver and peripheral tissues. On the other hand, the activated HEK-ProINS-Tf, with potentially stronger binding affinity to IR as evidenced by the stronger Akt phosphorylation and glucose production activity (Figure 4 and Figure 5), may immediately bind to IR after the conversion in the hepatocytes and subsequently be degraded before release to the blood circulation. Therefore, the glucose-lowering effect of HEK-ProINS-Tf is more selective in the liver, while ExpressTec-ProINS-Tf would possess additional effects on peripheral tissues. The results obtained following subcutaneous injection of HEK-ProINS-Tf compared to ExpressTec-ProINS-Tf (Figure 6a,b) support this hypothesis.

Tf has been considered as a drug carrier for improving oral absorption of protein therapeutics. The oral delivery study in this report also demonstrated that ExpressTec-ProINS-Tf, like other Tf-based fusion proteins [12,13], displayed a promising oral bioactivity. Based on the results in Figure 7, the blood glucose curve of orally administered ExpressTec-ProINS-Tf at a dose of 800 nmol/kg was very similar to that of subcutaneously injected ExpressTec-ProINS-Tf at 22.5 nmol/kg. This result suggested that the oral bioactivity of ExpressTec-ProINS-Tf was approximately 2–3% of subcutaneous injection. In the future, more experiments will be performed to further characterize the pharmacokinetic profiles of ExpressTec-ProINS-Tf. Additionally, some formulations, such as enteric-coated capsules and hydrogels, can be applied to further improve the oral bioavailability [18].

## 4. Materials and Methods

### 4.1. Development of Plasmid Construct

Both the amino acid sequence of ProINS and Tf protein were back translated into a nucleotide sequence with the codon algorithms. Both gene sequences were synthesized by Integrated DNA Technologies (IDT, Coralville, IA, USA). Plasmids were constructed at Ventria Bioscience Inc.

### 4.2. Rice genetic Transformation

#### 4.2.1. Particle Bombardment Transformation

Prior to particle bombardment transformation, linear DNA fragments containing the minimal expression cassettes were prepared by restriction enzyme digestion and subsequent cassette DNA purification. This process removes extraneous plasmid DNA and thus excludes any vector backbone sequence. These ‘clean’ expression cassette DNA fragments were used for biolistic particle transformation. The biolistic transformation was performed with the Biolistic PDS-1000/He system (Bio-Rad, Hercules, CA, USA) as previously described [10].

#### 4.2.2. Polymerase Chain Reaction (PCR) Analysis of Transgenic Plants with ProINS-Tf Gene

The PCR analysis of the regenerated plants was conducted using the Extract-N-Amp Plant PCR kit (Sigma-Aldrich, St. Louis, MO, USA). A pair of PCR primers located within the ProINS-Tf expression cassette were used for PCR amplification screening of positive transgenic plants. The amplification reaction condition was as follows: 94 °C for 5 min, 35 cycles of 94 °C, 1 min; 60 °C 1 min; and 72 °C for 1 min followed by 72 °C for 10 min. The PCR products were resolved in 1% agarose gel by electrophoresis.

#### 4.2.3. Plant Culture in Greenhouse

The PCR-positive transgenic plants were then transferred to greenhouse and planted into 6.5 × 6.5 cm pots containing a mix of 50% commercial soil, Sunshine #1 (Sun Gro Horticulture Inc., Agawam, WA, USA) and 50% natural soil from rice fields. The temperatures in the greenhouse were maintained at 30 °C during day time and 25 °C during night time. Fertilizer, water and pest management were carried out according to good agricultural practices to ensure healthy growth of the transgenic rice plants to maximize the chance of getting transgenic seeds.

### 4.3. Expression Analysis of Recombinant ProINS-Tf Protein from Transgenic Rice

Eight R1 seeds from each transgenic event were randomly picked, dehusked, and placed into eight wells within the same column of a 96-deep-well plate. Two hundred and fifty microliters of PBS were dispensed into each well to generate extracted protein which was pooled and analyzed for expression by immune-assays. The seed protein extracts from positive transgenic plants identified by the immuno-assay were resolved on a 4–20% Tris–glycine SDS–PAGE gel (Invitrogen, Carlsbad, CA, USA), electro-blotted onto a 0.45 μm nitrocellulose membrane for 1 h at 100 V in a Bio-Rad Protean System (Bio-Rad, Hercules, CA, USA). It was estimated that approximately 2 mg of ExpressTec-ProINS-Tf could be extracted from 100 g of the transgenic rice flour.

### 4.4. Expression and Purification of ProINS-Tf from HEK293 Cells

HEK-ProINS-Tf was expressed and purified as previously described [1]. In brief, HEK 293 cells (ATCC, Manassas, VA, USA) were transiently transfected with DNA plasmids containing the fusion gene of preproinsulin-Tf-6xHis via polyethylenimine-mediated transfection. The conditioned CD293 medium (Thermo Fisher Scientific Inc., Waltham, MA, USA) was collected 8-days post-transfection. The secreted HEK-ProINS-Tf was concentrated using tangential flow filtration (Millipore, Billerica, MA, USA) and then purified using nickel nitrilotriacetic acid agarose (Thermo Fisher Scientific Inc.). It was estimated that approximately 0.2 mg of HEK-ProINS-Tf could be produced from 1 L of the culture medium of transfected HEK293 cells.

### 4.5. TfR-Mediated Conversion Study in H4IIE Cells

A conversion study was performed to confirm the in vitro activation of ExpressTec-ProINS-Tf and to compare the conversion rates of both ProINS-Tfs. In this study, H4IIE rat hepatoma cells (ATCC) were treated with ProINS-Tfs from rice or HEK293 cells, in the presence or absence of 1000-fold excess of apo-Tf (Sigma), followed by incubation at 37 °C for 12 h. At the indicated time points, cell culture media were collected and subjected to insulin-specific radioimmunoassay (RIA, Millipore) that has less than 0.2% cross-reactivity with human proinsulin. The insulin concentrations of media were determined by insulin-specific RIA according to the manufacturer’s instructions, and the ^125^I radioactivity was counted using a gamma counter (Packard, Downers Grove, IL, USA).

### 4.6. Phosphorylation of Akt

To characterize the in vitro insulin-like bioactivity, Akt phosphorylation, was applied as a measurement of insulin signaling pathway activation. H4IIE cells were treated with DMEM *w*/0.1% bovine serum albumin (BSA; Sigma) for 18 h for serum starvation. The serum-deprived cells were then treated with human insulin (Sigma), ExpressTec-ProINS-Tf, HEK-ProINS-Tf, or DMEM *w*/0.1% BSA. At the specific time points, cells were lysed with cell extraction buffer (Invitrogen) supplemented with protease inhibitor cocktail (Sigma) and phenylmethanesulfonyl fluoride (Sigma). The cell lysates were subjected to Western blot analysis using anti-phospho-Akt (Ser 473) antibody (#4060, Cell Signaling Technology, Danvers, MA, USA) and anti-GAPDH antibody (#5174, Cell Signaling Technology, Danvers, MA, USA). The Western blots were developed using Amersham ECL Plus kits (GE Health care, Piscataway, NJ, USA), and quantified using Quantity One 1-D Analysis software (Bio-Rad, Hercules, CA, USA).

### 4.7. Inhibition of Glucose Production in H4IIE Cells

H4IIE cells in 24-well plates were treated with serial dilutions of human insulin, ExpressTec-ProINS-Tf, HEK-ProINS-Tf or rice-derived Tf (Invitria, Junction City, KS, USA) in serum-free DMEM at 37 °C for 24 h. After the treatment, the dosing solution was replaced with glucose production medium consisting of serum-, glucose- and phenol red-free DMEM supplemented with 2 mM sodium pyruvate and 40 mM sodium DL-lactate, followed by incubation at 37 °C for 3 h. The medium was harvested and glucose concentration of each sample was measured using the Amplex Red Glucose/Glucose Oxidase Kit (Invitrogen). The cells were then lysed with 1N NaOH, and total cellular protein was determined using protein bicinchoninic acid assay for normalization.

### 4.8. Type 1 Diabetes Mouse Model

The animal experimental protocol was approved by the Institutional Animal Care and Use Committee (IACUC) at the University of Southern California (ID: 11599, date of issuing: 1/24/2013). All animal studies were performed according to guidelines from National Institutes of Health. Male C57BL/6J mice (6-week old) purchased from The Jackson Laboratory (Bar Harbor, ME, USA) were used for pharmacodynamics studies. The mice were housed on a 12-h day and 12-h night cycle with room temperature maintained at 22 ± 3 °C and relative humidity at 50 ± 20%, and fed with a standard laboratory rodent diet (Labdiet, Richmond, IN, USA).

Type 1 diabetes of C57BL/6J mouse model was induced using high-dose STZ (150 mg/kg, Sigma) as previously described [2]. After 6-h fasting of mice, freshly prepared STZ solution in 100 mM sodium citrate buffer, pH 4.5 was intraperitoneally injected to induce diabetes. After waiting for 3–5 days for diabetes induction, mice with non-fasted blood glucose (BG) levels higher than 300 mg/dL were used for experiments. Blood samples were collected from the tail and blood glucose levels were measured using OneTouch Ultra glucose meter (LifeScan, Milpitas, CA, USA; detection range: 20–600 mg/dL).

### 4.9. In Vivo Glucose-Lowering Efficacies via Subcutaneous Injection

For the in vivo studies on free-fed diabetic mice, the experiments were carried out three days after STZ induction to avoid blood glucose levels beyond the detection range. The mice were subcutaneously injected with prepared protein samples. Mice had free access to water and food during the recording period. For the studies under fasting condition, the experiments were performed on day 5 after STZ induction. The mice were fasted from 2 h prior to injection, and remained fasted until 12 h post-administration. Mice were given ad libitum access to water under fasting condition. The blood glucose levels were measured from tail veins at the indicated time points.

### 4.10. In Vivo Glucose-Lowering Efficacies via Oral Administration

For studying the glucose-lowering effect of ExpressTec-ProINS-Tf via oral administration, the experiments were conducted on day 5 following STZ induction. The diabetic mice were fasted for 3 h for gastric emptying, and then the prepared proteins or buffer were administrated using an oral gavage needle or subcutaneous injection. Mice were kept fasting with free access of water until 12 h post-administration time point. The blood glucose levels were measured from tail veins at the indicated time points.

## 5. Conclusions

We demonstrated the feasibility of using a rice expression system for the production of ExpressTec-ProINS-Tf fusion protein. It is most likely that this expression system is also suitable for the production of other Tf-based fusion proteins. The in vitro and in vivo studies suggest that ExpressTec-ProINS-Tf possesses some minor different properties from HEK-ProINS-Tf, such as absence of glycosylation and slightly lower IR binding affinity. However, ExpressTec-ProINS-Tf still shares a great similarity with HEK-ProINS-Tf in their molecular characteristics and bioactivities. The oral delivery study further demonstrated the oral glucose-lowering effect of ExpressTec-ProINS-Tf. Thus, this low-cost ExpressTec-ProINS-Tf, with the advantages of oral absorption, liver targeting and activation, and long-term glucose-lowering effect, could be a potential candidate for the development of basal insulin replacement therapy.
